# Rock cavity nesting as the norm: Breeding songbirds of the temperate High Andes

**DOI:** 10.1002/ecy.3931

**Published:** 2023-01-03

**Authors:** Tomás A. Altamirano, Devin R. de Zwaan, Davide Scridel, Scott Wilson, Kathy Martin

**Affiliations:** ^1^ Department of Forest and Conservation Sciences University of British Columbia Vancouver British Columbia Canada; ^2^ Audubon Americas National Audubon Society Pucón Chile; ^3^ Cape Horn International Center (CHIC) Puerto Williams Chile; ^4^ CNR‐IRSA National Research Council – Water Research Institute Brugherio Italy; ^5^ Environment and Climate Change Canada Pacific Wildlife Research Centre Vancouver British Columbia Canada

**Keywords:** breeding sites, High Andes, mountain birds

Organisms living and breeding in alpine habitats must cope with severe environmental challenges such as temperature extremes, storms, resource limitations and, sometimes, hypoxia, resulting in short windows and reduced opportunities to reproduce (Chamberlain et al., 2023; Martin, 2001; Martin et al., 2017; Martin & Wiebe, 2004). Alpine habitats are defined as the area above the climatic treeline where vegetation growth is limited to cold‐tolerant grasses and forbs, low‐lying shrubs, or small patches of stunted trees <3 m in height, resulting in less diverse niche space relative to below the treeline (Körner, 2012; Körner et al., 2011). Despite these constraints, as well as limited habitat availability above treeline relative to the total landmass (Nagy & Grabherr, 2009; Testolin et al., 2020), ~12% of bird species breed in alpine habitats (de Zwaan et al., 2022a).

Globally, our understanding of avian nesting biology is limited, with clutch size and nest structure documented for only 53% and 45% of species, respectively (Reynolds & Deeming, 2015). Additionally, nest descriptions tend to be biased toward low‐elevation, Northern Hemisphere communities, with clear knowledge gaps for alpine breeding birds in south‐temperate mountains. Here, we describe the nest and breeding traits of songbirds breeding above treeline in the temperate High Andes, many of which previously lacked detailed descriptions. We also provide natural history notes on breeding phenology, parental care, and resource competition to provide a baseline for future studies to build on within these data‐deficient communities (Table 1). Finally, we compare the predominance of rock cavity‐nesting species above treeline in the temperate Andes with other temperate alpine songbird communities using a global alpine breeding bird dataset (de Zwaan et al., 2022a, 2022b) and generate a hypothesis framework for investigating variation in nest traits among alpine communities.

**TABLE 1:** Nest and breeding traits for High‐Andean birds in temperate mountains of southern Chile. Values represent the arithmetic mean ± standard deviation, with the range in parentheses for number of entrances, clutch size, and number of nestlings.

	Species				
Habitat attribute or breeding trait^a^	*G. rufipennis* (*n* = 7 nests)	*C. oustaleti* (*n* = 8)	*M. maclovianus* (*n* = 3)	*M. albilora* (*n* = 8)	*P. cyanoleuca* (*n* = 24)
Rock cavity					
No. entrances (no.)	1.3 ± 0.5 (1–2)	1.4 ± 0.7 (1–3)	1.3 ± 0.6 (1–2)	1.3 ± 0.5 (1–2)	1.0 ± 0.2 (1–2)
Height (m)	0.7 ± 1.1	0.2 ± 0.4	2.5 ± 3.5	1.7 ± 2.0	2.2 ± 1.6
Aspect (°)	185.8 ± 106.3	197.3 ± 107.9	266.0 ± 14.1	211.6 ± 109.5	158.7 ± 104.4
Entrance width (cm)	31.7 ± 25.4	16.8 ± 8.8	…	14.5 ± 5.6	33.4 ± 36.6
Entrance height (cm)	10.4 ± 1.5	6.8 ± 2.3	…	13.5 ± 6.8	7.1 ± 2.9
Horizontal cavity depth (cm)	74.9 ± 16.4	59.1 ± 13.4	…	42.8 ± 6.9	46.1 ± 16.7
Vertical cavity depth (cm)	9.1 ± 2.5	6.8 ± 2.4	…	17.3 ± 10.0	9.3 ± 5.0
Entrance concealment (%)^b^	2.9 ± 5.7	25.0 ± 34.7	20.0 ± 28.3	3.3 ± 8.2	7.2 ± 13.9
Habitat (1 m radius from nest)					
Distance to nearest vascular plant (cm)	18.9 ± 20.5	50.2 ± 75.4	40.0	6.5 ± 11.7	37.7 ± 66.7
Rock cover (%)^c^	73.3 ± 10.8	76.1 ± 20.5	90.0 ± 13.2	73.6 ± 15.2	82.0 ± 12.2
Shrub cover (%)	13.3 ± 4.1	6.9 ± 7.5	1.7 ± 2.9	12.0 ± 12.5	11.8 ± 9.7
Grass cover (%)	3.3 ± 2.6	7.0 ± 8.3	3.3 ± 3.0	2.2 ± 2.9	1.6 ± 2.6
Fern cover (%)	1.0 ± 2.0	0.0 ± 0.0	0.0 ± 0.0	1.2 ± 2.2	2.1 ± 4.7
Bare ground cover (%)	22.0 ± 34.8	10.0 ± 12.0	5.0 ± 8.7	44.4 ± 46.4	6.7 ± 21.2
Slope (°)	61.1 ± 27.8	48.0 ± 29.9	90.0 ± 0.0	63.7 ± 30.7	83.3 ± 27.0
Breeding parameters					
Clutch size	…	3.7 ± 0.6 (2–4)	…	2.8 ± 0.5 (2–3)	3.4 ± 0.7 (2–4)
No. nestlings	…	3.5 ± 0.7 (2–4)	…	2.5 ± 0.6 (2–3)	3.2 ± 0.8 (2–4)
Breeding success (%)^d^	100 (*n* = 4)	60 (*n* = 5)	…	80 (*n* = 6)	91 (*n* = 11)
Breeding behavior					
Which adult builds the nest?	F, M	F, M	F^e^	F^e^	…
Which adult incubates the eggs?	…	F, M	…	…	F, M
Which adult feeds the nestlings?	F, M	F, M	F, M	F, M	F, M
Building trip rate (trips/min)	0.3 ± 0.3	0.1 ± 0.1	0.04 ± 0.01	0.3 ± 0.1	0.5
Food provisional rate (trips/min)	0.2 ± 0.0	0.1 ± 0.1	0.3	0.2 ± 0.0	0.3 ± 0.1
Food items delivered^f^	Coleoptera, Hemiptera	Insects, Larvae	Lepidoptera, Larvae	Insects	…
Flush distance (m)	…	0.8 ± 1.3	…	…	0.0 ± 0.0
Incubation recess (min)	12	15.7 ± 4.0	…	39	13.0 ± 5.7
Breeding phenology					
Month of the first egg detected^g^	November	November	December	November	December
Second breeding attempt detected	Yes	Yes	Not detected	No	No

Abbreviations: F, female; M, male.

^a^ See Appendix S1 for detailed variable measurements.

^b^ Percentage of the entrance hidden by rocks or vegetation from the front of the cavity.

^c^ Covers (%) were estimated visually within a 1 m radius circle, up to 100% for each nest. Thus, the arithmetic means across nests do not necessarily sum to 100%.

^d^ Percentage of successful nests with sample size in parentheses. This sample size is a subset of the total described nests because not all nests could be monitored regularly for breeding success.

^e^ Only one adult, probably a female, was observed building the nest.

^f^ Food type is classified to the most detailed possible taxonomy and development stage.

^g^ With possible breeding earlier.

We located and monitored the nests of High‐Andean or alpine breeding bird communities in the temperate Andes over two breeding seasons (November–January, 2017 and 2018) across five volcanoes within the La Araucanía Region of southern Chile (39° S, 71° W). We monitored 50 nests between 1300–1800 m above sea level for five species: *Geositta rufipennis* (rufous‐banded miner, Furnariidae, *n* = 7 nests), *Cinclodes oustaleti* (gray‐flanked cinclodes, Furnariidae, *n* = 8), *Muscisaxicola maclovianus* (dark‐faced ground‐tyrant, Tyrannidae, *n* = 3), *Muscisaxicola albilora* (white‐browed ground‐tyrant, Tyrannidae, *n* = 8), and *Pygochelidon cyanoleuca* (blue‐and‐white swallow, Hirundinidae, *n* = 24). All nests were within rock cavities (Figure 1). Despite considerable search effort, we did not locate any ground nests. Rock cavity reuse was infrequent but recorded for *G. rufipennis* (*n* = 2 nests), *C. oustaleti* (*n* = 1), and *P*. *cyanoleuca* (*n* = 6) within and between breeding seasons (Table 1). See Appendix S1 for detailed information on the study site, methods, and variable measurements for Table 1.

**FIGURE 1:**
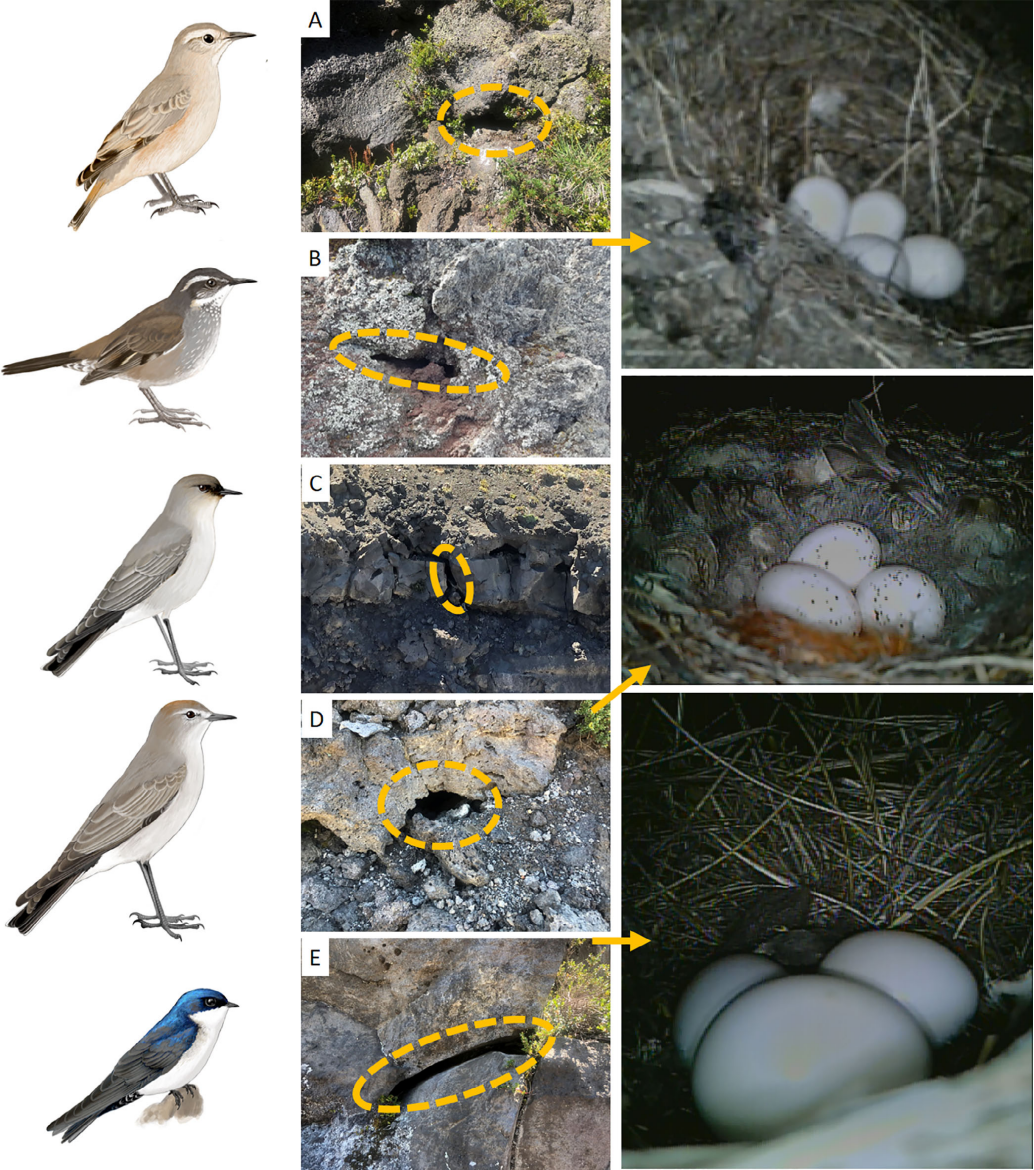
Rock cavity nests and eggs of: (A) *Geositta rufipennis* (rufous‐banded miner), (B) *Cinclodes oustaleti* (gray‐flanked cinclodes), (C) *Muscisaxicola maclovianus* (dark‐faced ground‐tyrant), (D) *Muscisaxicola albilora* (white‐browed ground‐tyrant), and (E) *Pygochelidon cyanoleuca* (blue‐and‐white swallow). Illustrations by Daniel Martínez. Photograph credits: Tomás A. Altamirano and Devin R. de Zwaan.

*Geositta rufipennis* occupied the deepest rock cavities of the five focal species (Table 1), such that we were unable to observe the nest contents. This species nested primarily in open rock fields or scree and was one of the earliest species to initiate breeding. Nest materials consisted of down feathers, grass, small twigs, fur, and rootlets. *Cinclodes oustaleti*, another Furnariid, used narrower cavities in younger volcanic rock (Table 1). Nests were built closer to the entrance, primarily using grass with minimal other materials, including one cigarette. The nests of *G. rufipennis* and *C. oustaleti* lacked a defined cup structure, resembling a loosely assembled platform.

*Muscisaxicola maclovianus*, a Tyranid ground‐tyrant, placed their nests mainly above rivers or streams in rock crevices (Table 1). Due to accessibility issues, we were unable to record all breeding parameters for *M. maclovianus*, but they used feathers and fur to insulate nests. *Muscisaxicola albilora* also nested in cliff crevices but was not associated with water (Table 1). Their nests consisted primarily of grass and rootlet cups lined with feathers. Despite being sister species, *M. maclovianus* and *M. albilora* appeared to have partitioned their nesting habitat niche, as both species were observed nesting or feeding frequently in the same general area, but never in close proximity.

*Pygochelidon cyanoleuca* nested in rock cavities with dimensions that varied widely among individuals relative to the other species (except perhaps *G. rufipennis*; Table 1), potentially indicating less reliance on a specific cavity shape. Nest cups consisted of broad‐leaved grasses or sedges, with limited instances of feathers or rootlets and, in two cases, toilet paper. This species was the most colonial, with large numbers nesting in the same rock face.

We recorded multiple instances of interspecific competition at nest sites. Specifically, *M. albilora* was highly aggressive toward *P. cyanoleuca*, nesting in similar sites despite frequent territorial disputes, with aggressive exclusion behavior between species near rock cavity entrances. In contrast, *M. maclovianus* was tolerant of *P. cyanoleuca* and often co‐associated in semicolonial aggregations. Less frequently, we observed competitive interactions between *M. albilora* and *Melanodera xanthogramma* (yellow‐bridled finch), and between *M. maclovianus* and *G. rufipennis*. This behavior may suggest that rock cavities are a limiting factor for High‐Andean bird populations, similar to tree cavity‐nesting birds (Cockle et al., 2010). In the Italian Alps, similar competition for rock and artificial cavities (i.e., snowfinch nest boxes, ski lift pylons, buildings) has been documented among *Montifringilla nivalis* (white‐winged snowfinch), *Phoenicurus ochruros* (black redstart), and *Motacilla alba* (white wagtail; Brambilla et al., 2019). Here, cavities experience within‐season interspecific reuse (i.e., between early and late broods), as well as species turnover in occupancy across years. In contrast, despite evidence of competition, we did not observe cavity takeover or interspecific reuse in the High‐Andean community. Another potential limiting resource is insulative nesting materials, which can improve nest success in alpine habitats (de Zwaan & Martin, 2018). We observed both *Leptastenura aegithaloides* (plain‐mantled tit‐spinetail) and *G. rufipennis* taking nesting material from an active *C. oustaleti* nest. Nest material kleptoparasitism is rarely reported outside of colonial‐nesting species (Slager et al., 2012), and never before at high elevations.

At the microhabitat scale, all species nested in proximity to vegetation, predominantly *Gaultheria pumila* (closest for all species), *Berberis empetrifolia, Cardamine chilensis*, *Chiliotrichum diffusum*, *Empetrum rubrum*, *Maytenus disticha*, ferns, and grasses. Breeding success was relatively high across species but varied considerably (60%–100%; Table 1). Other than clutch size and provisioning rate, parental behaviors such as incubation recesses and food provisioned varied among species, potentially reflecting differences in phenology (i.e., peak in November or December) and the stage of offspring development (Table 1). Ample opportunities exist for future research to address variation in cavity dimensions, plant associations, and parental behaviors within and among species to investigate potential consequences for reproductive success.

Using a global dataset of alpine breeding birds (de Zwaan et al., 2022a, 2022b), we found that the proportion of passerines nesting in rock cavities was greater in the southern Andes (this study site; 54%; 13 of 24 species) than in other major temperate alpine communities globally (excluding species that breed above treeline rarely or incidentally). In the southern ranges of Europe (e.g., Alps, Pyrenees, Carpathians), 39% (12 of 31) of alpine breeding passerines nest in rock cavities, compared with 24% (9 of 38) in the coastal and Rocky Mountains of North America, and 22% (26 of 117) on the Qinghai‐Tibetan Plateau. In the New Zealand Alps, which occur at a similar latitude to the temperate Andes, only one of four (25%) alpine passerine species nest in rock cavities.

The alpine breeding bird community in the southern Andes consists of ecologically and evolutionarily distinct species relative to lower elevations and other alpine communities globally (Altamirano et al., 2020; Martin et al., 2021). The underlying factors that drive the predominance of rock cavity‐nesting behavior in alpine breeding birds of the temperate Andes is an intriguing question that could yield insights into the evolution and maintenance of life‐history strategies in these rapidly changing environments (de Zwaan, Barras, et al., 2022). Here, we outline several nonmutually exclusive hypotheses that may explain the particularly high proportion of rock cavity nesters in the temperate Andes.

1. *Evolutionary history*. Avian communities above and below the treeline are more similar phylogenetically, or more closely related, in the temperate Andes than in the north temperate mountains of the Americas (Martin et al., 2021). Much of this similarity is driven by speciose families like Tyrannidae, which underwent rapid diversification relatively recently (~6 mya), about the same time as the Andes completed their uplift (Fjeldså et al., 2018). Given the high proportion of tree cavity‐nesting birds below the treeline in the region (57%; 29 species; Altamirano et al., 2017), it is plausible that cavity‐nesting species radiated into alpine habitats during a period of rapid niche diversification (Martin et al., 2021).
2. *Cavity availability and snow dynamics*. Temperate alpine habitats are strongly seasonal, with deep snow cover during winter (Nagy & Grabherr, 2009). In the southern Andes, rock ridges and outcrops are common due to recent and continuing volcanic activity, together with fluvial and glacial erosion. Rock substrates are the first to be exposed in spring as solar radiation warms the rock under the snow, contributing to faster snowmelt. Thus, in addition to potentially greater availability of rock cavities than in other temperate alpine habitats, rock cavities are also available earlier than the surrounding substrate, and thus may be more likely to be selected as a nesting resource especially given the short breeding seasons at high elevations (Altamirano et al., 2015).
3. *Exposure to severe conditions*. During the breeding season, relative humidity is low (~20%), temperatures are extreme and can fluctuate widely (Martin et al., 2017), and solar radiation is particularly strong in the High Andes (López‐Angulo et al., 2020). Dehydration can be detrimental to egg viability, and UV‐B radiation can damage developing embryos (Lahti & Ardia, 2016). Rock cavity nests may offer more benign microclimates to raise offspring, retaining greater levels of humidity, more consistent temperatures, and reducing UV‐B incidence compared with exposed alpine nests (Potapov, 2004).
4. *Predation risk*. Nest predation is often high in passerines and can be a strong selective force. While there is little information on nest predation rates in alpine habitats, particularly in the Southern Hemisphere, raptors and mammalian carnivores are the main predators above the treeline in the temperate Andes, with birds making up ~9%–17% of the diet of High‐Andean canids and felids (Walker et al., 2007; Zúñiga et al., 2020). Most of these mesopredators cannot access subterranean nests, potentially driving selection for rock cavity nests. Interestingly, however, for two nests (*C. oustaleti* and *P. cyanoleuca*), we observed ants preying upon early‐stage nestlings and potentially causing nest failure, suggesting other possible predation pressures in this system.

Temperate mountains and alpine habitats represent 5.5% and 2.6% of the global landmass excluding Antarctica and Greenland, respectively (Nagy & Grabherr, 2009; Testolin et al., 2020). These relatively small, isolated habitats and their associated biodiversity are also threatened by a rapidly warming climate and the potential for extirpation resulting from upslope, climate‐driven range shifts (Freeman et al., 2018; Scridel et al., 2018). We highlight the unique breeding biology of alpine passerines in the temperate Andes, underlining the importance of documenting basic nest traits and breeding parameters for data‐deficient and climate‐sensitive communities. Improving our knowledge of life‐history traits for these species is a prerequisite to understanding species resilience and the future of populations under climate change.

## CONFLICT OF INTEREST

The authors declare no conflict of interest.

## Supporting information


Appendix S1.

## Data Availability

Data (de Zwaan et al., 2022b) are available in Figshare at https://doi.org/10.6084/m9.figshare.20556750.

## References

[ecy3931-bib-0001] Altamirano, T. A. , D. R. de Zwaan , J. T. Ibarra , S. Wilson , and K. Martin . 2020. “Treeline Ecotones Shape the Distribution of Avian Species Richness and Functional Diversity in South Temperate Mountains.” Scientific Reports 10: 18428. 33116173 10.1038/s41598-020-75470-2 PMC7595238

[ecy3931-bib-0002] Altamirano, T. A. , J. T. Ibarra , M. de la Maza , S. A. Navarrete , and C. Bonacic . 2015. “Reproductive Life‐History Variation in a Secondary Cavity‐Nester across an Elevational Gradient in Andean Temperate Ecosystems.” The Auk 132: 826–35.

[ecy3931-bib-0003] Altamirano, T. A. , J. T. Ibarra , K. Martin , and C. Bonacic . 2017. “The Conservation Value of Tree Decay Processes as a Key Driver Structuring Tree Cavity Nest Webs in South American Temperate Rainforests.” Biodiversity and Conservation 26: 2453–72.

[ecy3931-bib-0004] Brambilla, M. , D. Scridel , B. Sangalli , F. Capelli , P. Pedrini , G. Bogliani , and D. Rubolini . 2019. “Ecological Factors Affecting Foraging Behaviour during Nestling Rearing in a High‐Elevation Species, the White‐Winged Snowfinch (*Montifringilla nivalis*).” Ornis Fennica 96: 142–51.

[ecy3931-bib-0005] Chamberlain, D. C. , A. Lehikoinen , and K. Martin . 2023. Ecology and Conservation of Alpine Birds. Ecology and Biodiversity Series. Cambridge: Cambridge University Press.

[ecy3931-bib-0006] Cockle, K. L. , K. Martin , and M. C. Drever . 2010. “Supply of Tree‐Holes Limits Nest Density of Cavity‐Nesting Birds in Primary and Logged Subtropical Atlantic Forest.” Biological Conservation 143: 2851–7.

[ecy3931-bib-0007] de Zwaan, D. R. , A. G. Barras , T. A. Altamirano , A. Asefa , P. Gokhale , R. S. Kumar , S. Li, et al. 2022. “Global Bird Communities of Alpine and Nival Habitats.” In Ecology and Conservation of Mountain Birds, edited by D. Chamberlain , A. Lehikoinen , and K. Martin . Cambridge, UK: Cambridge University Press.

[ecy3931-bib-0008] de Zwaan, D. R. , and K. Martin . 2018. “Substrate and Structure of Ground Nests Have Fitness Consequences for an Alpine Songbird.” Ibis 160: 790–804.

[ecy3931-bib-0009] de Zwaan, D. R. , D. Scridel , T. A. Altamirano , P. Gokhale , R. S. Kumar , C. S. Sevillano‐Ríos , A. G. Barras , et al. 2022a. “GABB: A Global Dataset of Alpine Breeding Birds and their Ecological Traits.” Scientific Data 9: 627. 36243729 10.1038/s41597-022-01723-6 PMC9569320

[ecy3931-bib-0010] de Zwaan, D. R. , D. Scridel , T. A. Altamirano , P. Gokhale , R. S. Kumar , C. S. Sevillano‐Ríos , A. G. Barras , et al. 2022b. “GABB: Global Alpine Breeding Bird Database.” Figshare. 10.6084/m9.figshare.20556750. PMC9569320 36243729

[ecy3931-bib-0011] Fjeldså, J. , J. I. Ohlson , H. Batalha‐Filho , P. G. P. Ericson , and M. Irestedt . 2018. “Rapid Expansion and Diversification into New Niche Space by Fluvicoline Flycatchers.” Journal of Avian Biology 49: jav‐01661.

[ecy3931-bib-0012] Freeman, B. G. , M. N. Scholer , V. Ruiz‐Gutierrez , and J. W. Fitzpatrick . 2018. “Climate Change Causes Upslope Shifts and Mountaintop Extirpations in a Tropical Bird Community.” Proceedings of the National Academy of Sciences of the United States of America 115: 11982–7. 30373825 10.1073/pnas.1804224115 PMC6255149

[ecy3931-bib-0013] Körner, C. 2012. Alpine Treelines: Functional Ecology of the Global High Elevation Tree Limits. Berlin: Springer.

[ecy3931-bib-0014] Körner, C. , J. Paulsen , and E. M. Spehn . 2011. “A Definition of Mountains and their Bioclimatic Belts for Global Comparisons of Biodiversity Data.” Alpine Botany 121: 73–8.

[ecy3931-bib-0015] Lahti, D. C. , and D. R. Ardia . 2016. “Shedding Light on Bird Egg Color: Pigment as Parasol and the Dark Car Effect.” American Naturalist 187: 547–63. 10.1086/685780 27104989

[ecy3931-bib-0016] López‐Angulo, J. , D. S. Pescador , A. M. Sánchez , A. L. Luzuriaga , L. A. Cavieres , and A. Escudero . 2020. “Impact of Climate, Soil and Biotic Interactions on the Interplay of the Different Facets of Alpine Plant Diversity.” Science of the Total Environment 698: 133960. 31493573 10.1016/j.scitotenv.2019.133960

[ecy3931-bib-0017] Martin, K. 2001. “Wildlife in Alpine and Sub‐Alpine Habitats.” In Wildlife‐Habitat Relationships in Oregon and Washington, edited by D. H. Johnson and T. A. O'Neil , 285–310. Corvallis, OR: Oregon State University Press.

[ecy3931-bib-0018] Martin, K. , T. A. Altamirano , D. R. de Zwaan , K. G. Hick , A. Vanderpas , and S. Wilson . 2021. “Avian Ecology and Community Structure across Elevation Gradients: The Importance of High Latitude Temperate Mountain Habitats for Conserving Biodiversity in the Americas.” Global Ecology and Conservation 30: e01799.

[ecy3931-bib-0019] Martin, K. , and K. L. Wiebe . 2004. “Coping Mechanisms of Alpine and Arctic Breeding Birds: Extreme Weather and Limitations to Reproductive Resilience.” Integrative and Comparative Biology 185: 177–85. 10.1093/icb/44.2.177 21680497

[ecy3931-bib-0020] Martin, K. , S. Wilson , E. C. MacDonald , A. F. Camfield , M. Martin , and S. A. Trefry . 2017. “Effects of Severe Weather on Reproduction for Sympatric Songbirds in an Alpine Environment: Interactions of Climate Extremes Influence Nesting Success.” The Auk 134: 696–709.

[ecy3931-bib-0021] Nagy, L. , and G. Grabherr , eds. 2009. The Biology of Alpine Habitats, 1st ed. Oxford: Oxford University Press.

[ecy3931-bib-0022] Potapov, R. L. 2004. “Adaptations of Birds to Life in High Mountains in Eurasia.” Acta Zoologica Sinica 50: 970–7.

[ecy3931-bib-0023] Reynolds, S. J. , and D. C. Deeming . 2015. “Incubating New Ideas about Avian Reproduction.” In Nests, Eggs, and Incubation: New Ideas about Avian Reproduction, edited by D. C. Deeming and S. J. Reynolds , 1–7. Oxford: Oxford University Press.

[ecy3931-bib-0024] Scridel, D. , M. Brambilla , K. Martin , A. Lehikoinen , A. Iemma , A. Matteo , S. Jähnig , et al. 2018. “A Review and Meta‐Analysis of the Effects of Climate Change on Holarctic Mountain and Upland Bird Populations.” Ibis 160: 489–515.

[ecy3931-bib-0025] Slager, D. L. , M. E. McDermott , and A. D. Rodewald . 2012. “Kleptoparasitism of Nesting Material from a Red‐Faced Spinetail (*Cranioleuca erythrops*) Nest Site.” Wilson Journal of Ornithology 124: 812–5.

[ecy3931-bib-0026] Testolin, R. , F. Attorre , and B. Jiménez‐Alfaro . 2020. “Global Distribution and Bioclimatic Characterization of Alpine Biomes.” Ecography 43: 779–88.

[ecy3931-bib-0027] Walker, R. S. , A. J. Novaro , P. Perovic , R. Palacios , E. Donadio , M. Lucherini , M. Pia , and M. S. López . 2007. “Diets of Three Species of Andean Carnivores in High‐Altitude Deserts of Argentina.” Journal of Mammalogy 88: 519–25.

[ecy3931-bib-0028] Zúñiga, A. H. , J. R. Rau , V. Fuenzalida , and A. Fuentes‐Ramírez . 2020. “Temporal Changes in the Diet of Two Sympatric Carnivorous Mammals in a Protected Area of South–Central Chile Affected by a Mixed–Severity Forest Fire.” Animal Biodiversity and Conservation 43: 177–86.

