# Supplementary material for: Rock cavity nesting as the norm: Breeding songbirds of the temperate High Andes
Source: Ecology. 2023 Jan 3;104(2):e3931. doi: 10.1002/ecy.3931 (PMC10078528; doi:10.1002/ecy.3931)
Supplement: Supplementary file 1 — Appendix S1. [file ECY-104-0-s001.pdf]

Appendix S1. Methods

Journal Name: Ecology
Title. Rock cavity nesting as the norm: Breeding songbirds of the temperate High Andes
Authors: Tomás A. Altamirano, Devin R. de Zwaan, Davide Scridel, Scott Wilson & Kathy Martin

Study area

We searched for and monitored nests from alpine breeding bird communities on five volcanos in the La Araucanía Region, Chile (39°S, 71°W): Tolhuaca, Sollipulli, Sierra Nevada, Llaima, and Rukapillan. Alpine habitats begin above treeline and extend to the perennial snowline (nival belt), which in our high-Andean study area, encompasses an elevational range of 1,300 to 1,800 m. During the breeding season (November-January), environmental conditions are characterized by high winds (average 2.0 m/s), severe, fluctuating temperatures (-6.1 to 38.4 °C), low relative humidity (average 55.8%), and low barometric pressure (average 848.4 mbar). High Andean habitats are dominated by a mix of stunted woody shrubs, herbaceous plants, tussock (*Poa*) grasses, volcanic rocks, and vertical canyon walls shaped by fast-flowing streams. The main vascular plant species present in these habitats are Chaura (*Gaultheria pumila*), Magellan Barberry (*Berberis microphylla*), Heath Barberry (*Berberis empetrifolia*), and Quinchamali (*Quinchamalium chilense*) (see Figure S1).

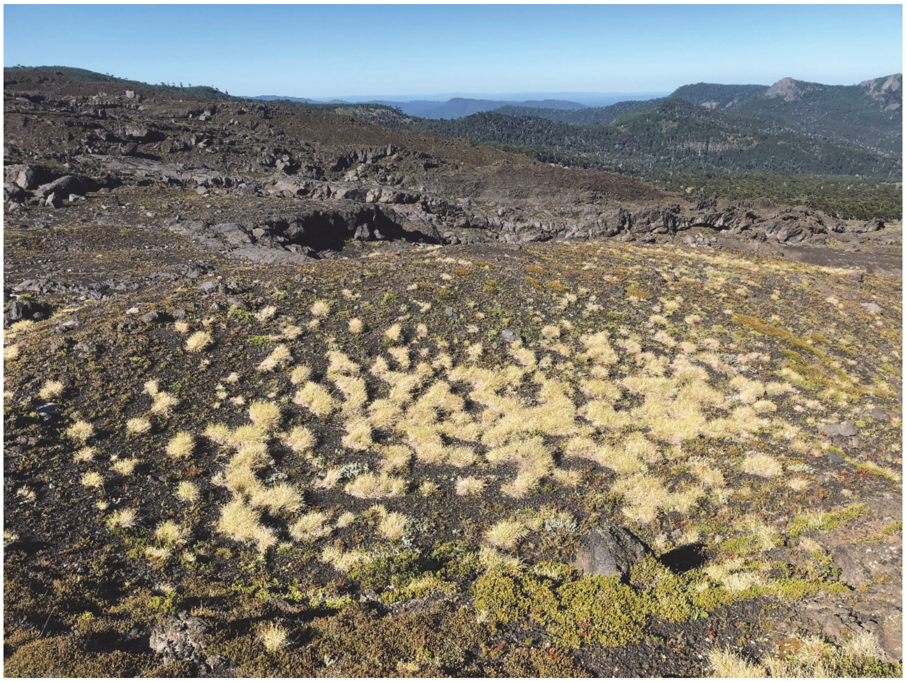

Figure S1. High Andean habitat above treeline in the south temperate mountains of Chile. Photo Credit: Tomás A. Altamirano.

Breeding survey, cavity, and nest traits

We located nests based on behavioral observations and systematic territory searches above treeline. Behavioral cues included nest building, incubation bouts, or nestling provisioning (Martin et al. 2017). Once we found a nest, we checked the interior of the cavity for evidence of nesting activity and nesting status using a flashlight and hand-held mirror, or a 2 m-long endoscope (RIDGID CA-350; Altamirano et al. 2017). Cavities were considered actively used if it contained at least one egg or nestling (Altamirano et al. 2015). For cavities where nest contents could not be observed, nesting status was determined by observing parental behavior. For each nest, we recorded bird species, as well as clutch size and/or brood size (i.e., number of nestlings) when possible. We monitored nests regularly to determine nest fate, considering a nest successful when at least one nestling fledged. For a minimum of 15 minutes during each visit, we observed adult breeding behavior from a distance and behind a natural blind to limit observer effects. We recorded the number of nest building visits, the duration of incubation or recess bouts, the number of provisioning trips, and the type of food item brought to the nest. Food items were classified to the most detailed possible taxonomy and development stage when observable, and thus are typically biased towards larger food items. We recorded nest materials, when possible, either by inspecting the nest or observing nest building activities from a distance.

Following the completion of nesting activity, we measured cavity traits for each nest and classified the surrounding microhabitat. Specifically, we recorded: 1) number of entrances, 2) height of cavity entrance, 3) aspect, 4) entrance width and height, 5) horizontal and vertical cavity depth, and 6) concealment. *Number of entrances* was defined as the number of independent openings a bird could use to access the internal nest chamber. *Height of cavity entrance* was the distance from the ground to the lower lip of the cavity entrance. Ground in this context is the first horizontal surface (e.g., ledge, platform) from which a predator could feasibly stand on. *Aspect* was measured outward from the perspective of the main cavity entrance. *Entrance width and height* was the maximum horizontal and vertical distance, respectively, between edges of the main entrance. *Horizontal and vertical cavity depth* refers to the nest chamber, and as such was measured at the location of the nest inside the cavity. Finally, *concealment* was the percentage of the entrance hidden by rocks or vegetation from the front of the cavity.

At the micro-habitat level, we recorded distance to and species of the nearest vegetation, slope of the substrate where the cavity occurs (degrees, 0°: flat ground, 90°: vertical wall), and percent cover of rock, shrub, grasses, ferns, and bare ground within a 1 m radius of the cavity by intervals of 10% up to 100% for each nest. Rock cover represents large rock patches that could potentially support a cavity, while bare ground refers to smaller grain size, such as dirt and stones.
